# Role of Resin Microsphere Y90 Dosimetry in Predicting Objective Tumor Response, Survival and Treatment Related Toxicity in Surgically Unresectable Colorectal Liver Metastasis: A Retrospective Single Institution Study

**DOI:** 10.3390/cancers13194908

**Published:** 2021-09-29

**Authors:** Tina Sankhla, Bernard Cheng, Nariman Nezami, Minzhi Xing, Ila Sethi, Zachary Bercu, David Brandon, Bill Majdalany, David M. Schuster, Nima Kokabi

**Affiliations:** 1 Division of Interventional Radiology and Image-Guided Medicine, Department of Radiology and Imaging Sciences, Emory University, Atlanta, GA 30308, USA; tina.vijay.sankhla@emory.edu (T.S.); nariman.nezami@emory.edu (N.N.); minzhi.xing@jhmi.edu (M.X.); zachary.louis.bercu@emory.edu (Z.B.); bill.majdalany@emoryhealthcare.org (B.M.); 2 Morehouse School of Medicine, Atlanta, GA 30310, USA; bernard.paulkid.cheng@emory.edu; 3 Division of Nuclear Medicine and Molecular Imaging, Department of Radiology and Imaging Sciences, Emory University, Atlanta, GA 30308, USA; sila2@emory.edu (I.S.); david.brandon@emoryhealthcare.org (D.B.); dschust@emory.edu (D.M.S.); 4 Emory University Hospital Midtown, 550 Peachtree Street NE, Atlanta, GA 30308, USA

**Keywords:** unresectable colorectal liver metastasis, selective internal radiation therapy, objective tumor response, overall survival, treatment related toxicity

## Abstract

**Simple Summary:**

Colorectal liver metastases are difficult to treat, with only a minority of patients eligible for surgical resection. Yttrium-90 selective internal radiation therapy is an alternative treatment currently used for patients who have progressed on chemotherapy. A technique called dosimetry allows clinicians to analyze how much radiation was delivered to target lesions post-treatment. The aim of this study is to evaluate the relationship of various dosimetric parameters with objective tumor response, overall survival, and treatment related toxicity with the potential goal of optimizing Yttrium-90 treatment in this patient population. Additionally, other potential predictors of survival outcomes, including clinical and demographic factors, were also evaluated. We found that delivering a mean tumor dose ≥100 Gy when using resin microspheres was significantly associated with objective tumor response and prolonged overall survival. In this study, no mean non-tumoral liver dose threshold was found to predict treatment related toxicity.

**Abstract:**

Purpose: To Evaluate the correlation between tumor dosimetric parameters with objective tumor response (OR) and overall survival (OS) in patients with surgically unresectable colorectal liver metastasis (CRLM) undergoing resin-based Ytrrium-90 selective internal radiation therapy (Y90 SIRT). Materials and Methods: 45 consecutive patients with CRLM underwent resin-based Y90 SIRT in one or both hepatic lobes (66 treated lobes total). Dose volume histograms were created with MIM Sureplan^®^ v.6.9 using post-treatment SPECT/CT. Dosimetry analyses were based on the cumulative volume of the five largest tumors in each treatment session and non-tumoral liver (NTL) dose. Receiver operating characteristic (ROC) curve was used to evaluate tumor dosimetric factors in predicting OR by Response Evaluation Criteria for Solid Tumors at 3 months post-Y90. Additionally, ROC curve was used to evaluate non-tumoral liver dose as a predictor of grade ≥ 3 liver toxicity and radioembolization induced liver disease (REILD) 3 months post Y90. To minimize for potential confounding demographic and clinical factors, univariate and multivariate analysis of survival with mean tumor dose as one of the factors were also performed. Kaplan-Meier estimation was used for OS analysis from initial Y90 SIRT. Results: 26 out of 45 patients had OR with a median OS of 17.2 months versus 6.8 months for patients without OR (*p* < 0.001). Mean tumor dose (TD) of the five largest tumors was the strongest predictor of OR with an area under the curve of 0.73 (*p* < 0.001). Minimum TD, and TD to 30%, 50%, and 70% of tumor volume also predicted OR (*p*’s < 0.05). Mean TD ≥ 100 Gy predicted a significantly prolonged median OS of 19 vs. 11 months for those receiving TD < 100 Gy (*p* = 0.016). On univariate analysis, mean TD < 100 Gy, presence of any genomic mutation, presence of MAPK pathway mutation, bilobar hepatic metastases and diffuse metastatic disease (>10 lesions per liver lobe) were found to be predictors of shorter median OS. On multivariate analysis, mean TD < 100 Gy, presence of any genomic mutation, and diffuse hepatic metastatic disease were found to be independent predictors of shorter OS. Overall, six (13.3%) patients developed grade ≥ 3 liver toxicity post Y90 of whom two (4.4%) patients developed REILD. No dose threshold predicting grade ≥ 3 liver toxicity or REILD was identified. Conclusions: Mean TD ≥ 100 Gy in patients with unresectable CRLM undergoing resin-based Y90 SIRT predicts OR and prolonged OS.

## 1. Introduction

Colorectal cancer is the second leading cause of cancer-related mortality in the USA, with metastatic disease to the liver often the ultimate cause of death [1]. While surgical resection is the only potentially curative treatment in approximately 20% of surgical candidates, many of these patients are not surgical candidates by the time of diagnosis [2]. Y90 has been extensively studied in patients with surgically unresectable colorectal liver metastasis (CRLM) and proven to be well tolerated and provide favorable survival benefits in appropriately chosen patients, including patients who have received multiple lines of prior chemotherapy and/or prior resection [3,4,5,6,7,8]. Many recent studies are aimed at further defining the patient population that would receive the most benefit. When comparing chemotherapy plus SIRT versus chemotherapy alone as a first line treatment, studies showed decreased incidence of disease progression within the liver, which did not translate to a benefit in overall survival [9]. Accordingly per NCCN guidelines for CRLM, Y90 SIRT is currently used only in the salvage setting in the population who have had disease progression while on one or more lines of chemotherapy [10]. Recent and ongoing studies are aiming to further delineate the ideal timing and patient characteristics for a more personalized approach to treatment decision making [11,12]. 

The manufacturer recommended activity for resin microspheres is based on the body surface area (BSA) model, which uses body surface area as a predictor of theoretical liver volume with compensation for increasing tumor burden [13]. However, the BSA model does not consider the actual activity and distribution delivered to the liver and to tumoral tissue, which is in part related to tumor vascularity. Additionally, it does not take into account the lung shunt fraction, and the volume of the lobe or segment to be treated. To maximize the efficacy of Y90 SIRT, tumoricidal effects needs to be optimized while minimizing overall radiation to non-target tissue to allow effective treatment with minimal treatment related toxicities including radiation pneumonitis and radioembolization induced liver disease (REILD). The benefits of such personalized dosimetry have been proven for hepatocellular carcinoma (HCC) through emerging evidence [14], but yet more data is needed for metastatic disease where the multifocal nature of the disease adds more layers of complexity. This added challenge can now be addressed by several commercially available dosimetry softwares with capability of semi-automated contouring of the liver and segmenting of the tumors.

This study aimed to evaluate the correlation between various tumor dosimetric parameters and objective tumor response (OR) as well as overall survival (OS) in patients with surgically unresectable colorectal liver metastases (CRLM) treated with resin based Ytrrium-90 selective internal radiation therapy (Y90 SIRT). Furthermore, non-tumoral liver (NTL) dose as a predictor of moderate to severe liver toxicity, REILD and survival was investigated in this study.

## 2. Results

### 2.1. Baseline Demographic Characteristics

The studied population demographic characteristics are summarized in Table 1. Overall, 45 patients with CRLM underwent resin based Y90 SIRT in one or both hepatic lobes, with a total of 66 treated lobes included. Of these patients, 26 were over the age of 65 and 19 were under 65. There were 29 males and 16 females. While an ECOG score of >2 is generally considered a contraindication to liver directed therapy, our cohort included 2 patients with an ECOG score of 3 on the basis of wheelchair bound status due to remote amputation from non-cancer related causes and were deemed appropriate candidates for Y90 RE by multidisciplinary evaluation. All patients had an ECOG score of less than or equal to 3, with 27 patients with an ECOG of 0. All patients had genomic testing of the primary tumor, and 25 (56%) of the patients had a genomic mutation with 21 (47%) of those patients have a mutation involving mitogen-activated protein kinase (MAPK) pathway. Additionally, 32 (71%) of patients had diffuse (>10 Intrahepatic Tumors/hepatic lobe) metastatic liver disease and 25 (56%) had also extrahepatic metastatic disease. For additional baseline clinical data, please refer to Table 1. 

### 2.2. Tumor Response and Overall Survival

**Figure 1:**
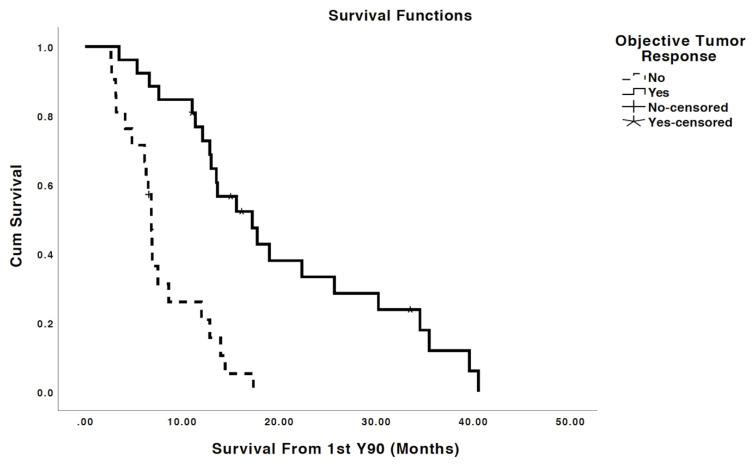
Patients exhibiting objective response had a median OS of 17.2 months vs. 6.8 months for those without OR (*p* < 0.001).

Median OS was 12.9 months from the time of first Y90 treatment. Overall, 26 (55%) patients had OR at 3 months post Y90 SIRT and 21 (45%) patients did not have OR per Response Evaluation Criteria in Solid Tumors (RECIST) version 1.0. Patients with OR had a significantly prolonged OS of 17.2 months from the time of first Y90 SIRT versus 6.8 months for the patients without OR (*p* < 0.001) (Figure 1). 

### 2.3. Tumor Dosimetry, Dose Response Thresholds, Implication on Survival

**Figure 2:**
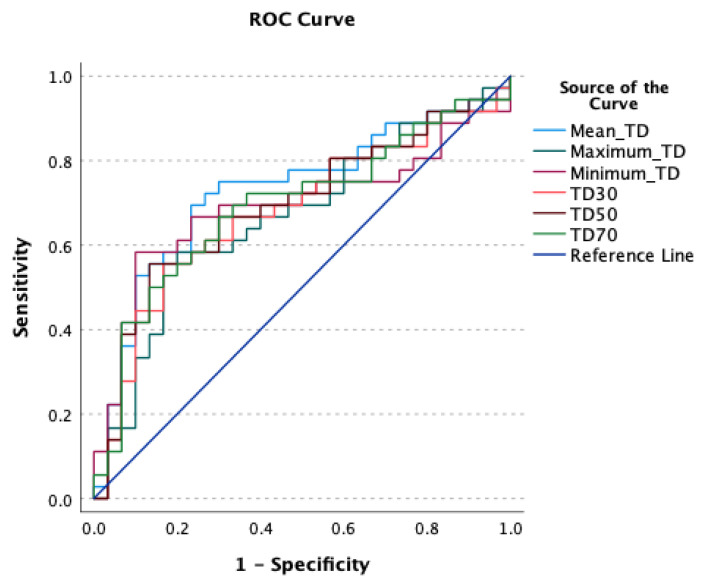
ROC Curve for dosimetry parameters predicting partial or complete response based on Response Evaluation Criteria in Solid Tumors with the following AUC’s: Mean TD (0.730), TD 30 (0.679), TD 50 (0.690), TD 70 (0.692), maximum TD (0.692), minimum TD (0.666).

**Figure 3:**
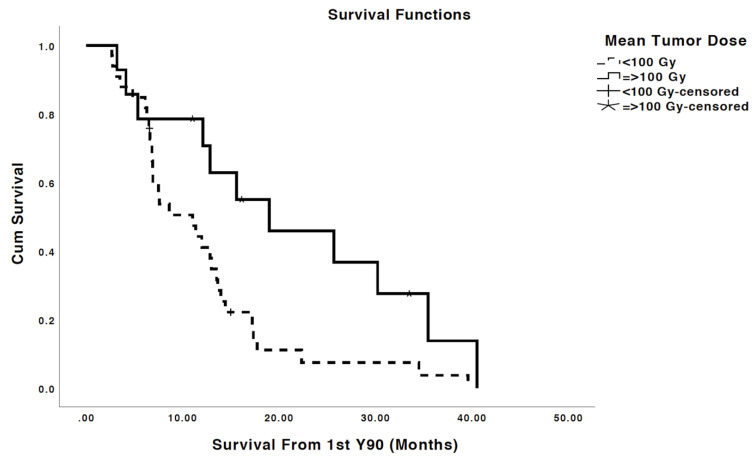
Patients who received mean TD ≥ 100 Gy had a median OS of 19 months compared to 11 months for those receiving less (*p* = 0.016).

Average minimum, mean, and maximum tumor dose delivered to treated and analyzed tumors were 35.3 Gy, 68.7 Gy and 94.1 Gy, respectively (Table 2). Mean tumor dose (TD) was the strongest predictor of OR with ROC analysis yielding in AUC of 0.73 (*p* < 0.001). ROCs also showed that TD 30%, TD 50%, TD 70%, and TD 99% (Minimum TD) of tumor volume predicted OR (*p*’s < 0.05) with the AUC’s of 0.679, 0.69, 0.692, and 0.692, respectively (Figure 2). Additionally, patients with mean TD ≥ 100 Gy had a significantly prolonged median OS of 19 months versus 11 months for patients who received TD < 100 Gy (*p* = 0.016; Figure 3).

**Figure 4:**
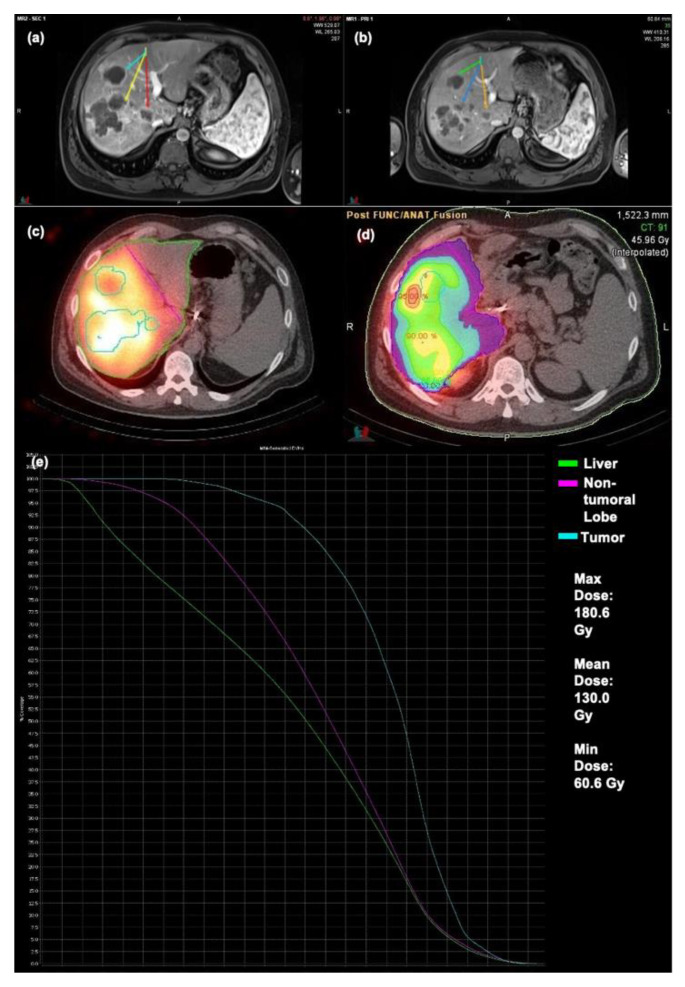
Patient exhibiting OR to Y90 therapy (**a**) Pre-op MRI (**b**) Post-op MRI (**c**) Bremsstrahlung SPECT/CT (**d**) Dosimetry map (**e**) DVH Curve.

An example of a patient exhibiting OR can be seen in Figure 4. Prescribed activity of 42.8 mCi of resin microspheres was administered to the patient’s replaced right hepatic artery. Follow-up MRI demonstrated a partial response to treatment for the treated lobe. Treated lesions in this case had a mean TD of 130.0 Gy, a maximum TD of 180.6 Gy, and a minimum TD of 60.6 Gy.

**Figure 5:**
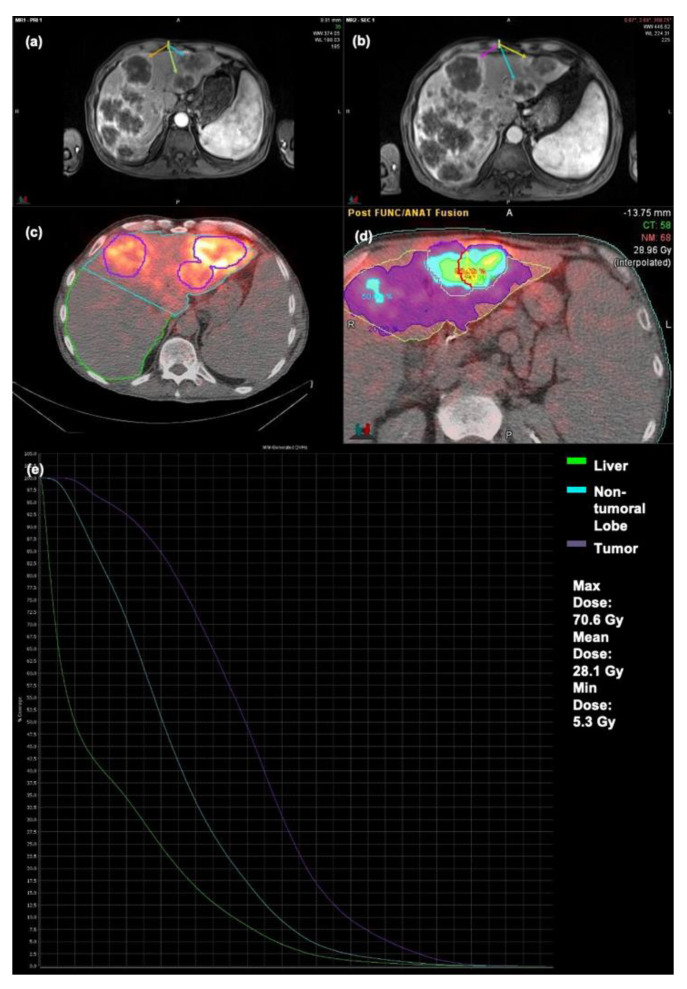
Patient failing to show OR following Y90 therapy (**a**) Pre-op MRI (**b**) Post-op MRI (**c**) Bremsstrahlung SPECT/CT (**d**) Dosimetry map (**e**) DVH Curve.

An example of a patient not showing OR can be seen in Figure 5. An amount of 20.6 mCi of resin microspheres were injected into the left hepatic artery. Subsequent MRI showed tumor progression within prior tumors increasing in size and new tumors being identified. Tumors for this patient had a mean TD of 28.1 Gy, a maximum TD of 70.6 Gy, and a minimum TD of 5.3 Gy.

### 2.4. Other Predictors of Survival

Using log-rank analysis, other potential demographic and clinical factors affecting survival were evaluated (Table 3). Presence of any genomic mutation, mutation involving MAPK pathway, bilobar hepatic metastasis, and diffuse hepatic metastasis were found to be predictors of prolonged median OS from first Y90 (Table 3).

On MVA of the clinical factors that were found to be predictors of prolonged survival using long-rank analysis (UVA), presence of any genomic mutation and diffuse disease found to be independent predictors of shorter OS from the time of first Y90 SIRT (Table 4). Furthermore, mean TD ≥ 100 Gy was found to be an independent predictor of prolonged OS (RR = 0.245, *p* < 0.001).

### 2.5. NTL Dose Implication on Toxicity and Survival

**Figure 6:**
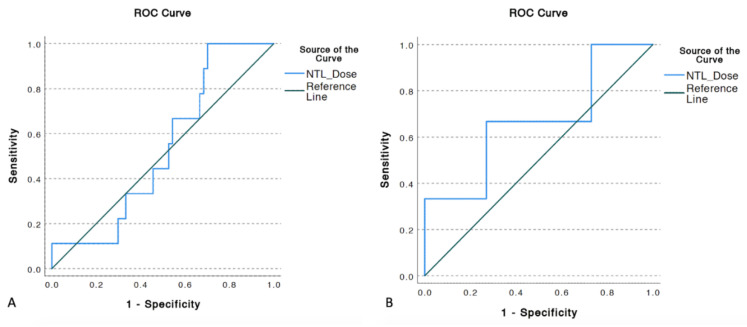
ROC curve for evaluation of relation of mean NTL dose and development of (**A**) CTCAE grade ≥ 3 liver toxicity (AUC: 0.532, *p* = 0.712) and (**B**) REILD (AUC: 0.667, *p* = 0.349) demonstrated mean NTL dose to not be a statistically significant predictor for either outcome.

**Figure 7:**
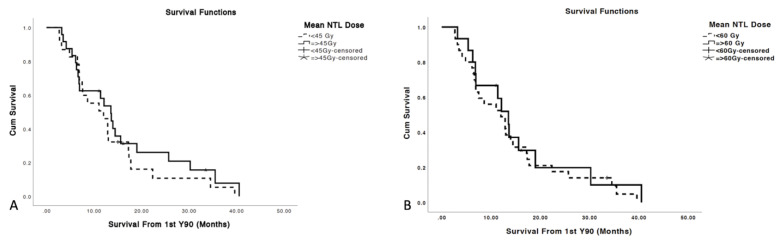
Mean NTL dose thresholds of 45 Gy (**A**) and 60 Gy (**B**) did not predict prolonged or shorter OS from the time of first Y90 radioembolization, *p*-values 0.401 and 0.547, respectively.

Overall, 6 (13.3%) patients developed grade ≥ 3 liver toxicity post Y90, of whom 2 (4.4%) patients developed REILD, which in this study was classified as the development of ascites or jaundice in the absence of biliary obstruction within 8 weeks of Y90 RE in the absence of progression of intrahepatic tumors [15]. Of the remaining 4 patients, 3 patients had a resolution of grade ≥ 3 liver toxicity and 1 patient had stable toxicity at 1-year post Y90 radioembolization. No statistically significant dose threshold predicting grade ≥ 3 liver toxicity or REILD was identified on ROC curve analysis (AUC’s < 0.5, *p*-values > 0.05) (Figure 6). Furthermore, no difference in OS was observed for patients with mean NTL dose greater than or less than 45 Gy and 60 Gy (Figure 7). 

## 3. Discussion

Our findings showed that patients with OR and the mean TD ≥100 Gy had significantly prolonged median OS, respectively improving OS for 10.4 and 8 months (*p* < 0.001 and *p* = 0.016, respectively). Tumor dose response threshold and its implication on survival has been extensively investigated in the setting of HCC treated with both glass- and resin-based Y90 microspheres [16,17,18,19]. There is now level one evidence demonstrating target tumor dose of >205 Gy to be a predictor of OR and prolonged OS [14]. However, due to the generally multifocal nature of metastatic disease in the liver and complexity of contouring, dosimetry analysis of these diseases treated with Y90 SIRT has remained under-investigated with only a few previously published studies outlined below. With the advent of semi-automated commercially available dosimetry softwares, dosimetry analysis for liver metastasis treated with Y90 SIRT is now easily achievable. In this study we demonstrated that, similar to HCC, there is a TD response threshold for surgically unresectable CRLM treated with Y90 SIRT.

The results of this study were congruous with those of prior studies in showing a significantly prolonged overall survival in patients with objective response by RECIST following Y90 treatment compared to patients without objective response by RECIST [20,21]. Hence, determining tumor dose thresholds to achieve objective tumor response is of paramount importance to improve patients’ outcomes. Aside from OR, other imaging characteristics have also been shown to be predictive of outcomes in CRLM treated with Y90 SIRT [22]. For example, a phase III clinical research trial with 10 patients found that a tumor-to-normal ratio cutoff of 1 on MAA shunt studies could predict metabolic response [23]. 

With the intention of establishing a better understanding of what treatment parameters can predict objective response, the previously described dosimetric factors were then analyzed. Results showed that the strongest predictor of tumor response by RECIST criteria, as well as prolonged survival, was the mean cumulative tumor dose of 5 largest targeted tumors in each liver lobe. Specifically, the mean tumor dose >100 Gy predicted prolonged overall survival. 

Few prior retrospective and prospective studies have shown a dose-response relationship for CRLM treated with Y90 SIRT. A retrospective study of 24 patients, which utilized post-Y90 PET/CT in patients with chemorefractory CRLM treated with resin microspheres, used the partition-based dosimetry model to demonstrate mean absorbed dose cutoffs of 39 Gy predicting non-metabolic response on PET and 60 Gy predicting high metabolic response [24]. The study also demonstrated that cases where all tumors received a TD > 39 Gy were associated with OS of 13 months vs. 5 months for those who had at least one tumor with TD < 39 Gy [24]. A prospective study conducted between 2011–2014 with 30 patients, which used the BSA model for treatment planning, reported a conservative estimate of at least 40–60 Gy tumor dose was predictive of metabolic response [25], and another study found 50 Gy (using modified BSA method) as threshold to achieve objective response [26], both using resin microspheres. A more recent 2021 retrospective study with 31 patients using glass microspheres found that median TD for complete responders was 196 Gy versus 177 Gy for partial responders [27]. This higher TD compared to our findings of 100 Gy can most likely be explained by their use of glass microspheres compared to our use of resin microspheres.

In our study, cumulative volume and Y90 distribution of the 5 largest tumors were used in each treated lobe, which is different than the prior studies where mean tumor dose was applied for each individual lesion. This may explain the higher dose response thresholds reported in our study. Interestingly, similar to the tumor dose threshold of 100 Gy that the current study’s results suggest for CLRM is similar to the threshold reported by previous dosimetry studies pertaining to HCC treated with resin microspheres [28,29,30]. Furthermore, these results correlate with recently published international expert consensus recommendations for personalization of SIRT for Y90 SIRT liver treatment which recommend a minimum mean target absorbed dose to tumor of 100–120 Gy for hepatocellular carcinoma, liver metastatic colorectal cancer and cholangiocarcinoma with moderate/strong expert agreement [31]. 

Currently, there is no robust data on NTL dose threshold that can accurately predict clinically significant liver toxicity post Y90. In our study, we did not identify a threshold of mean NTL dose that would predict development of moderate to severe liver directed toxicity or REILD which is discordant to the threshold of 40 Gy to the NTL recommended by the aforementioned expert consensus [31]. Additionally, no threshold of mean NTL to adversely affect overall survival from Y90 was identified in this study. 

While this study focused specifically on dosimetric factors that predicted OS, prior studies have identified baseline pre-treatment characteristics that predict response to Y90 RE. One such study which aimed to create a pre-treatment risk stratification nomogram identified CEA level, transaminase level, sum of 2 largest liver diameters, and no liver surgery before SIRT, to be independently associated with OS [32]. A subsequent study assessed 40 baseline characteristics using multivariate analysis, and again identified carcinoembryonic antigen, alanine aminotransferase level, and the sum of the 2 largest tumor diameters, and additionally identified tumor differentiation level and number of sites of extrahepatic disease, to be independently associated with overall survival [33]. While some factors are not redemonstrated across studies, for example, differing takes on the impact of surgery naïve liver as a predictor of OS [32,34], some factors, including CEA level and transaminase level, are consistently identified across numerous studies [32,33,34,35,36]. Multiple studies have also assessed imaging characteristics as predictors of OS. A study looking at a pre-treatment triphasic liver scan showed that tumor arterial enhancement fraction, which was defined as the arterial phase enhancement divided by the portal phase enhancement and can act as an estimate of the hepatic artery blood supply as a fraction of the total blood supply, was predictive of metabolic response on post-Y90 RE PET/CT [37]. 

Evaluating response to treatment based on post-treatment imaging has proved challenging. RECIST criteria has been the most commonly used way to assess post-treatment response. However, it comes with inherent weaknesses as response versus progression is solely assessed based on changes to the maximum diameter of target lesions which can be confounded by treatment-related edema and tumor necrosis which may erroneously make lesions appear larger in early post-treatment follow-up, and does not take into account other factors such as degree of enhancement and metabolic activity of the treated lesion [38,39,40,41]. There is data to suggest that the metabolic response based on changes in metabolic tumor volume and total lesion glycolysis on PET/CT is predictive of OS [42,43]. Changes in metabolic activity on PET/CT may be an earlier marker of response to Y90 RE and incorporation into follow-up protocol may provide additional value in subsequent clinical decision making [35]. 

The role of tumor genomics is another area of investigation with studies aiming to define what mutations may confer favorable response to Y90 RE. In one retrospective study of 40 patients, PI3K pathway mutation was identified as an independent predictor of longer time to local progression after RE [44]. In another retrospective study of 58 patients with genomic analysis prior to Y90 RE, there was a statistically significant prolonged OS in patients with MAPK wild type compared to mutation [11]. Further study is needed to validate these findings and define the role of genomic testing in the treatment algorithm.

In this study, we attempted to determine if any of the previously demonstrated clinical and demographic factors confound the survival improvement seen in patients with mean TD ≥ 100 Gy. We found that the presence of any genomic mutation in the primary tumor, presence of diffuse hepatic metastatic disease and the mean TD threshold of 100 Gy were all independent predictors of OS from first Y90 SIRT. 

This study was limited by several factors including its retrospective design. Additionally, the relatively small sample size may adversely affect the survival analysis observations related to tumor dosimetry and other clinical factors.

## 4. Materials and Methods

### 4.1. Study Design and Population

The Institutional Review Board approved retrospective single-institution multihospital study enrolled 45 consecutive patients with surgically unresectable CRLM who received resin based Y90 SIRT in one or both lobes after at least one line of traditional chemotherapy treatment failure from February 2013 to March 2018. The need for written consent was waived. The study was conducted at a tertiary referral center, and patients’ records were reviewed using institutional electronic medical records. Survival information was documented through a combination of electronic medical records, patient, patients’ family members, and public obituaries. Fifteen patients were excluded from this study due to having insufficient imaging files necessary for dosimetry or determining OR.

### 4.2. Ytrrium-90 Selective Internal Radiation Therapy (Y90 SIRT) Technique

Using previously described methods [16], all patients were treated with resin-based Y90 microspheres (Sirtex Medical Ltd., Woburn, MA, USA). Approximately 2 weeks prior to treatment, patients underwent technetium-99m macroaggregated albumin (Tc-99m MAA) shunt study, with approximately 4 mCi of Tc-99m MAA administered either from common hepatic artery or in right and left hepatic arteries in a split dose fashion, depending on distribution of tumor. Perfusion of the targeted tumors from a specific microcatheter position prior to injection was ascertained with intraprocedural cone beam CT during the mapping study. Y90 activity to be administered was calculated using body BSA model taking into account % tumor involvement as recommended by the SIR-Sphere package insert [13]. On the day of treatment, Y90 was administered in a lobar fashion using a 2.4–2.8 Fr microcatheter, and if the patient required bilobar treatment, the hepatic lobe with larger disease burden was treated first and the remaining lobe was treated 4–6 weeks following initial treatment. Dextrose 5% in water was used during the administration of Y90 in all patients to maximize dose delivery and minimize incidence of stasis as has been suggested by the results of prior studies [45]. No stasis during Y90 delivery was seen in any of the patients in this cohort.

### 4.3. Dosimetry Analysis

**Figure 8:**
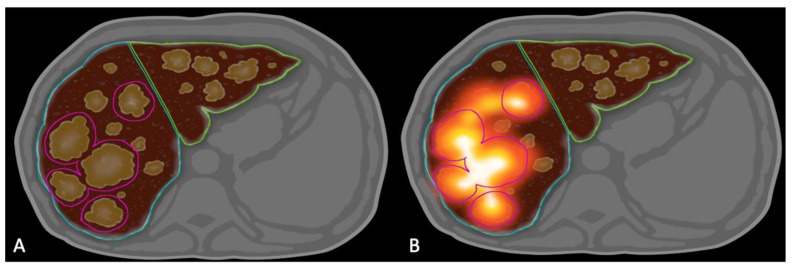
Dosimetry Method Diagram. (**A**) contour the target lobe and 5 largest tumors on the target lobe. (**B**) Bremsstrahlung SPECT/CT showing the cumulative activity in the 5 and 5 largest tumors in the target lobe.

At the time of treatment, Y90 SPECT/CT was obtained immediately following radioembolization for each patient. MIM Sureplan^®^ software v.6.9 (MIM Software, Cleveland, OH, USA) was used retrospectively to create a volumetric region of interest around the 5 largest tumors in each treated lobe using the semi-automated dosimetry module of the software. Contours around tumors and normal liver were created on the most recent contrast enhanced cross-sectional imaging (CT or MRI) before Y90 therapy (Figure 8). They were then fused to Y90 SPECT/CT images using MIM software. The dose to the cumulative tumor and NTL were then calculated manually and using the software-generated dose volume histograms (DVH). Mean NTL was calculated for each treated lobe. If a patient had bilobar treatment, an average of mean NTL for the 2 therapies were used for the analysis. 

### 4.4. Image Analysis

OR, defined as having either a complete or partial response, was determined via dynamic contrast enhanced multiphase MRI taken 3 months post-procedure using Response Evaluation Criteria in Solid Tumors (RECIST) 1.0 evaluating the cumulative response of 5 targeted tumors treated in each lobe. A complete response was defined as when targeted tumors are no longer seen. Partial response was defined as a ≥30% reduction in the total diameter of targeted tumors [46].

### 4.5. Treatment Related Toxicity

Moderate and severe Y90 related treatment related toxicity to the liver was evaluated using Common Terminology Criteria for Adverse Events (CTCAE) version 5 [47] at 3, 6 and 12 months post Y90 radioembolization. Grade ≥ 3 toxicities for alanine aminotransferase (ALT), aspartate aminotransferase (AST), total serum bilirubin, and serum albumin were recorded. Additionally, development of REILD was evaluated in the study cohort.

### 4.6. Variables and Definitions

The five largest tumors in each treated lobe were contoured. The dosimetry analysis was performed on the conglomerate (cumulative volume) of these tumors considering them as one large tumor. The following factors were then evaluated:

Mean TD: Mean dose delivered to all the tumor conglomerate in each lobe in Gray.
TD 30: Average of dose delivered to at least 30% of the conglomerate tumor volume in Gray.
TD 50: Dose delivered to at least 50% of the conglomerate tumor volume in Gray.
TD 70: Dose delivered to at least 70% of the conglomerate tumor volume in Gray.
TD 99: Dose delivered to at least 99% of the conglomerate tumor volume in Gray; i.e., minimum dose delivered to the tumors.
Maximum TD: Maximum dose delivered to the conglomerate tumor volume in Gray.
OR: Objective response by RECIST 1.0 Criteria in the 5 targeted tumors [46].
Overall survival: Survival from time of first Y90 SIRT.
Mean NTL Dose: Mean dose delivered to non-tumoral liver lobe in each treatment session.

### 4.7. Survival and Statistical Analysis

Receiver operating characteristic (ROC) curve was used to calculate the area under the curve (AUC) and evaluate the utility of each dosimetric factor in predicting OR by RECIST criteria at 3 months post Y90. Kaplan-Meier estimation was used for survival analysis from first Y90 SIRT. To minimize for potential confounding demographic and clinical factors affecting survival, univariate analysis (UVA) of mean tumor dose and other clinical factors were performed using log-rank analysis. Multivariate analysis (MVA) using Cox-regression performed on the significant factors identified by UVA.

ROC curve was also used to determine statistically significant mean NTL dose thresholds of developing moderate to severe treatment related liver toxicity and REILD. Log-rank analysis was then used to evaluate mean NTL dose as predictors of overall survival (OS) from first Y90 radioembolization. Statistical analysis was performed using SPSS version 25 (IBM Corporation, Armonk, NY, USA) with statistical significance of *p* < 0.05.

## 5. Conclusions

In conclusion, a mean tumor dose of >100 Gy into the largest five targeted tumors in a liver lobe treated with resin-based Y90 SIRT was found to be an independent predictor of objective tumor response and prolonged overall survival in patients with surgically unresectable CRLM. No non-tumoral liver dose threshold was identified to predict toxicity and survival outcomes.

## Figures and Tables

**Table 1:** Patient Baseline Characteristics.

Variables	Groups	Number	
Age	≥65	26	58%
	<65	19	42%
Gender	Male	29	64%
	Female	16	36%
ECOG	0	27	60%
	1	13	29%
	≥2	4	9%
	Unknown	1	2%
Pre-Op Lab Values	International Normalized Ratio	1.08	Range: 0.91–1.8
	Aspartate Aminotransferase	35.5 IU/L	Range: 21–78
	Alanine Aminotransferase	27.0 IU/L	Range: 17–59
	Total Bilirubin	0.68 mg/dL	Range: 0.41–1.81
	Creatinine	0.85 mg/dL	Range: 0.51–1.72
	Albumin	3.6 g/dL	Range: 2.9–4.8
	Sodium	137 mmol/L	Range: 131–148
Multifocal Disease	Yes	45	100%
	No	0	0%
Any Genomic Mutation	Yes	25	56%
	No	20	44%
MAPK Pathway Mutation	Yes	21	47%
	No	24	53%
Baseline Ascites	Yes	4	9%
	No	41	91%
Baseline CEA Level	>20 ng/mL	28	62%
	≤20 ng/mL	17	38%
CEA Decrease post Y90 SIRT	Yes	23	51%
	No	22	49%
Diffuse Disease (>10 Intrahepatic Tumors/hepatic lobe)	Yes	32	71%
	No	13	29%
Extra Hepatic Metastatic Disease	Yes	25	56%
	No	20	44%
Index Tumor Size	Mean	3.2 cm	
	Range	1.8–8.7 cm	
Index Tumor	≥5 cm	14	31%
	<5 cm	31	69%
Bilobar Intrahepatic Tumors	Yes	28	62%
	No	17	38%

ECOG: Eastern Cooperative Oncology Group Performance Status. MAPK: Mitogen-activated protein kinase. CEA: Carcinoembryogenic antigen.

**Table 2:** Statistics for dosimetry parameters.

Mean TD	Mean	68.7 Gy
	Range	18.1–744.5 Gy
Maximum TD	Mean	94.1 Gy
	Range	34.7–866.1 Gy
TD 30%	Mean	70.31 Gy
	Range	22.5–421.3 Gy
TD 50%	Mean	63.17 Gy
	Range	11.0–325.9 Gy
TD 70%	Mean	57.6 Gy
	Range	7.0–290.8 Gy
TD 99% (Minimum TD)	Mean	35.3 Gy
	Range	3.1–232.1 Gy

TD: Tumor Dose, Gy: Gray.

**Table 3:** Overall Characteristics and Median Overall Survival from Hepatic Metastasis.

Variables		Median OS (Months)	*p* Value
Any Mutation	No	15.4	**0.020**
	Yes	9.1	
MAPK	No	16.3	**0.001**
	Yes	8.4	
Age	≤65	14.2	0.201
	>65	12.1	
Sex	Male	11.6	0.212
	Female	13.8	
ECOG	0	15.1	0.077
	≥1	10.3	
Baseline Ascites	Yes	12.0	0.311
	No	13.5	
Baseline CEA level	>20 ng/mL	11.9	0.975
	≤20 ng/mL	12.7	
CEA decrease post Y90 RE	Yes	15.3	0.075
	No	11.1	
Bilobar Hepatic Metastases	Yes	10.2	**0.029**
	No	16.9	
Index lesion	≥5 cm	12.5	0.254
	<5 cm	12.8	
Diffuse Hepatic Metastases	Yes	9.2	**<0.001**
	No	18.1	
Extra-hepatic Metastases	Yes	11.9	0.961
	No	12.7	

Bolded values indicate statistical significance (*p* < 0.05).

**Table 4:** Multivariate Analysis of Prognostic Factors.

Factor	Relative Risk (RR)	*p*-Value	Standard Error
MAPK Mutation	1.137	0.502	0.559
Any Mutation	3.297	**0.027**	0.479
Mean TD ≥ 100 Gy	0.245	**<0.001**	0.443
Bilobar Disease	1.213	0.623	0.414
Diffuse Disease	3.219	**0.012**	0.421

Bolded values indicate statistical significance (*p* < 0.05).

## Data Availability

Anonymized study data are available from the corresponding author upon reasonable request.
